# Culture of Murine Embryonic Metatarsals: A Physiological Model of Endochondral Ossification

**DOI:** 10.3791/54978

**Published:** 2016-12-03

**Authors:** Dean A. Houston, Katherine A. Staines, Vicky E. MacRae, Colin Farquharson

**Affiliations:** ^1^Developmental Biology, The Roslin Institute and R(D)SVS, The University of Edinburgh

**Keywords:** Developmental Biology, Issue 118, Metatarsal, chondrocyte, extracellular matrix, mineralization, murine, endochondral ossification

## Abstract

The fundamental process of endochondral ossification is under tight regulation in the healthy individual so as to prevent disturbed development and/or longitudinal bone growth. As such, it is imperative that we further our understanding of the underpinning molecular mechanisms involved in such disorders so as to provide advances towards human and animal patient benefit. The mouse metatarsal organ explant culture is a highly physiological *ex vivo* model for studying endochondral ossification and bone growth as the growth rate of the bones in culture mimic that observed *in vivo*. Uniquely, the metatarsal organ culture allows the examination of chondrocytes in different phases of chondrogenesis and maintains cell-cell and cell-matrix interactions, therefore providing conditions closer to the *in vivo *situation than cells in monolayer or 3D culture. This protocol describes in detail the intricate dissection of embryonic metatarsals from the hind limb of E15 murine embryos and the subsequent analyses that can be performed in order to examine endochondral ossification and longitudinal bone growth.

**Figure:**
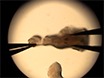


## Introduction

The skeleton is a highly intricate, dynamic and complex organ that has a range of functions spanning from locomotion, to ion homeostasis. Comprised of bone and cartilage, the skeleton consists of functionally distinct cell populations which operate to maintain it's structural, biochemical and mechanical integrity ^1^. One of the primary functions of the skeleton is its role in development and growth. These processes require the orchestration of multiple cellular populations which are regulated through tight endocrine and genetic control.

With the exception of the flat bones *e.g.* scapula, cranium and sternum, the mammalian skeleton is formed through the process known as endochondral ossification. This process, regulated both molecularly and spatially, begins with the condensation of mesenchymal cells derived primarily from the mesoderm ^2^. Under the influence of the transcription factor SOX9, cells in the interior of the condensations differentiate into chondrocytes, expressing and secreting cartilage specific extracellular matrix (ECM) components such as type II collagen and aggrecan. Cells at the margin of the condensation, express type I collagen and form the perichondrium, delineating the prospective bone ^3^. Later, the osteoprogenitors of the perichondrium differentiate into osteoblasts, directed by the expression of the transcription factors, Runx2 and osterix ^4^. This leads to a predominance of osteoblasts and this layer is renamed the periosteum which forms a mineralized bony collar around the mid-section of the bone. Vascular penetration of the periosteum and invasion of the cartilaginous scaffold occurs bringing with it, haematopoetically derived bone resorbing cells, osteoclasts, which resorb the chondrocyte remnants and much of the cartilaginous matrix ^5^. The spaces formed are filled by osteoprogenitors giving rise to the primary ossification center which progressively occupies the core of the diaphysis and merges with the periosteum. Other cells give rise to the bone marrow. Around birth, blood vessels and osteoprogenitors penetrate the cartilage rich matrix at either side (epiphysis) of the developing diaphysis to form the secondary ossification center. Situated between the epiphysis and diaphysis at either end of the long bones is a band of cartilage - the epiphyseal growth plate - which is responsible for coordinating linear growth of all long bones ^6^.

Chondrocytes within the growth plate initially proliferate and subsequently progress through morphologically distinct zones, coordinated by sequential expression of transcription and growth factors. The culmination of this sequence of events is the exit from the cell cycle and hypertrophy of chondrocytes at the center of this cartilage precursor. Hypertrophic chondrocytes strongly express type X collagen and matrix metalloproteinase-13 (MMP13) and their considerable volume enlargement in the direction of longitudinal growth provides the greatest contribution to bone growth in mammals ^7,8^. Furthermore, hypertrophic chondrocytes mineralize their surrounding ECM through the release of matrix vesicles, membrane bounded structures specialized for the production of amorphous calcium phosphate through their inclusion of phosphatases (tissue non-specific alkaline phosphatase (TNAP) and PHOSPHO1) and calcium channeling proteins (annexins) ^9-11^. This calcified cartilage is invaded by blood vessels from the underlying marrow resulting in osteoclast recruitment and matrix resorption. This is followed by osteoblast migration and alignment to the surface of the calcified cartilage remnants where they lay down a layer of osteoid which is rapidly mineralized. The woven bone of the primary spongiosa is later remodeled to lamellar bone of the secondary spongiosa ^12^. Epiphyseal fusion results in the cessation of endochondral ossification ^13^.

Endochondral ossification is under tight regulation by many endocrine and paracrine hormones and growth factors so as to prevent disturbed development and/or longitudinal bone growth ^14^. A better understanding of the molecular and cellular mechanisms underpinning these processes is therefore imperative in our pursuit of clinical advances towards human benefit. Previous research into the similarities and differences between growth plates of different ages, locations and species have greatly improved our understanding of the physiological mechanisms surrounding growth plate dynamics ^15,16^. However, the study of isolated chondrocytes is difficult, as removal of chondrocytes from their environment can lead to dedifferentiation, accompanied by loss of expression of key markers such as *Col2a1*^17^.

The mouse metatarsal organ explant culture, pioneered by Prof. Elisabeth Burger and colleagues in the Netherlands, is a highly physiological *ex vivo* model for studying endochondral ossification and bone growth as the growth rate of the bones in culture mimic that seen *in vivo *^18^. Uniquely, the metatarsal organ culture allows the examination of chondrocytes in different phases of chondrogenesis and maintains cell-cell and cell-matrix interactions, therefore providing conditions closer to the *in vivo* situation than cells in culture ^19-21^. Moreover, this model allows for direct examination of linear bone growth which is not possible in primary chondrocyte cultures. It also allows for the separation of systemic and local factors therefore permitting the specific analysis of the local effects on the growth plate.

The culture of metatarsals allows for the investigation of numerous parameters. Daily measurements of total length and of the mineralized regions of the rudiments can be performed with standard phase contrast microscopy and image analysis software. Histological, *in situ* hybridization and immunohistological staining may be readily performed on metatarsal sections, which allows for the spatial resolution of both cellular and molecular entities, a major advantage over traditional cell culture methods. The immunohistochemical approach includes the detection and quantification of 5-bromo-2'-deoxyuridine (BrdU) uptake by proliferating chondrocytes ^22^. Additionally, further processing of metatarsal tissues may be performed to allow for downstream analysis of mRNA expression (RT-qPCR), protein and intracellular signaling analysis (Western blotting) or lipid composition (mass spectrometry). Most studies to date use cultured embryonic metatarsals rather than metatarsals from post-natal animals. Whilst both models can be informative, the study of embryonic bones has several advantages: they grow faster and can be exploited to study the initiation and progression of the mineralization process ^23-25^.

This protocol describes in detail the intricate dissection of embryonic metatarsals from the hind limb of embryonic day 15 (E15) murine embryos. Furthermore, the growth and mineralization of anatomically distinct metatarsals is shown.

## Protocol

Procedures involving animal subjects were conducted in compliance with the U.K. Animals (Scientific Procedures) Act 1986 and animals were maintained in accordance with the Home Office published Code of Practice for the Housing and Care of Animals Bred, Supplied or Used for Scientific Purposes.

NOTE: Experiments carried out using wild-type C57Bl/6J mice are well established and described below. Embryonic metatarsals from different mouse strains may similarly be cultured *in vitro*, although their growth and mineralization rates may differ to those detailed herein.

### 1. Dissection Conditions and Preparation of Instruments

1. Perform all media preparation, tissues dissections and culture work in a laminar flow hood to ensure the sterility of media and embryonic rudiments.
2. Allow culture and dissection media to equilibrate for at least 1 hr in a 37 °C, 5% CO_2 _incubator prior to use.
3. Sterilize dissection scissors, Dumont #5 and Dumont #4 tweezers alongside a pair of superFine Vannas micro scissors (8 cm straight, 3 mm blades) by autoclaving. After autoclaving, immerse the dissection equipment in 70% ethanol prior to use.

### 2. Preparation of Dissection and Culture Media

1. **Prepare dissection medium**
  1. Dilute α-Minimum Essential Media (without nucleosides, αMEM) in phosphate buffered saline (PBS) (1:13) and resuspend bovine serum albumin (BSA) at 2 mg/m**l** before filter sterilizing through a 0.22 µm, 33 mm diameter syringe filter. NOTE: Dissection medium can be made, filter sterilized and stored in aliquots at -20 °C indefinitely.
2. **Prepare culture medium**
  1. Culture embryonic metatarsals in 300 µl of culture medium (in each well of a 24-well culture plate). Prepare the culture medium by adding 0.2% w/v BSA; 5 µg/ml L-ascorbic acid phosphate; 1 mM β-glycerophosphate (βGP; optional – see results section); 0.05 mg/ml gentamicin and 1.25 µg Amphotericin B to αMEM and filter sterilize through a 0.22 µm, 33 mm diameter syringe filter.

### 3. Murine Embryonic Metatarsal Dissection and Culture

1. Cull the pregnant mouse, harboring 15-day-old embryos, by cervical dislocation in accordance with home office guidelines in the U.K.
2. Place the animal supine and disinfect skin by spraying with 70% ethanol. Using dissection scissors make a large incision in the midline, penetrating both the skin and the peritoneum to expose the abdominal cavity.
3. Locate the two uterine horns of the animal in the dorsal region of the body cavity. Remove the uterine horns by firstly freeing each uterine from the mesometrium by careful incisions with dissection scissors and then finally cutting the uterine horns free at their base. Place uterine horns into dissection medium.
4. Separate each embryo by cutting between implantation sites along the uterine horn and remove the embryos from their individual sacs using forceps. Cull embryos by decapitation in accordance with home office guidelines in the U.K.
5. After disinfection of the skin by spraying with 70% ethanol, use Vannas micro scissors to remove the hind limbs from the embryos by incising around the proximal femur. This ensures as much of the hind limb as possible is removed. Place the hind limbs into a petri dish submerged in dissection medium prior to metatarsal dissection.
6. Under a dissecting microscope, begin to remove the skin surrounding the embryonic foot, by pinching and pulling it off using the #5 tweezers whilst holding the hind limb steady using the #4 tweezers. This is most effectively performed by grasping the skin at around the level of the tibia and pulling towards the phalanges. The use of scissors at this stage to cut the very thin skin is not required.
7. Carefully hold the tarsals of the embryonic foot with #4 tweezers and remove the first and fifth metatarsals by pinching off near the tarsals using #5 tweezers and discard.
8. With the foot held in the same position, use the #5 tweezers to disrupt the connective tissues between the three remaining phalanges and metatarsals. Insert the tip of #5 tweezers at the joint between the phalanges and metatarsals to remove the phalanges from the metatarsals.
9. Finally, use #5 tweezers to disrupt the connective tissues between the tarsals and metatarsals. Again place the tip of #5 tweezers in the joint space between the tarsals and metatarsals and gently free the metatarsals from the tarsals.
10. Gently pick up the now free metatarsals with #4 tweezers and place in fresh dissection medium. NOTE: Careful dissection should provide 6 metatarsals from each embryo.
11. When all required metatarsals have been dissected, carefully place metatarsals individually into the pre-warmed 24-well plates containing 300 µl of culture media. Culture metatarsals at 37 °C in a 5 % CO_2 _incubator for up to 14 days. NOTE: Do not change the media of E15 cultures until at least day 5 of culture as this affects their mineralization capability ^26^. The reasons for this are unclear but linear growth and matrix mineralization may be dependent upon stimulation by growth factors released from the metatarsals.

### 4. Analysis and Manipulation of Metatarsal Cultures

1. On the day of dissection (day 0) make initial longitudinal measurements using a microscope with a digital camera attached and image analysis software. Measure the length of the central mineralization zone when it appears after a few days of culture.
2. Periodically, as required, make further measurements (usually every 2^nd^ day) until the final day of culture at which point metatarsal bones are processed dependent upon required outcomes (discussed in introduction).
3. Supplement medium of metatarsal organ cultures with various exogenous factors *e.g.* growth factors, hormones and pharmacological agents from day 0 of culture^ 22,24^. In all studies of this kind, control untreated metatarsals are required. NOTE: Although it is advisable that the control bones are the equivalent rudiment from the contralateral leg, there have not been any differences in linear growth or mineralization potential of cultured E15 2^nd^, 3^rd^ and 4^th^ metatarsals (see below, **Figure 2**).

## Representative Results

The aim of this method was to isolate embryonic metatarsals and culture them for examination of longitudinal bone growth and ECM mineralization. Using our protocol described herein, embryonic metatarsal bones were successfully dissected, as visualized by light microscopy. Measurements of metatarsal bones, conducted as indicated in **Figure 1**, showed their capacity to grow in longitudinal length and mineralize their ECM (**Figures 2 **and **3**). Mineralization of the matrix is first noted in the mid-diaphysis of the bone rudiment and is easily observed by light microscopy. No staining is required although the uptake of calcein may offer enhanced visualization of newly formed mineral.

Studies utilizing embryonic metatarsals to date ^22,27,28^ have pooled the dissected 2^nd^, 3^rd^ and 4^th^ (central three) metatarsals, assuming *in vitro* growth and mineralization to be comparable. *In vivo* developmental of murine metatarsals, however, reveals that the 3^rd^ and 4^th^ digits have evidence of mineralization prior to all other metatarsals and phalanges ^29^. Assessment of the growth and mineralization potential of individually isolated and cultured E15 2^nd^, 3^rd^ and 4^th^ metatarsals revealed no significant differences in the appearance or extent of mineralization after 7 days of culture (**Figure 2**). No difference in the percentage increase from baseline was found during the culture period. As no differences in mineralization potential was noted the standard protocol of pooling the 2^nd^ 3^rd^ and 4^th^ metatarsals appears valid for future studies.

Traditionally, investigators have added βGP to media used to culture embryonic metatarsals to promote ECM mineralization. However, in cell culture studies, it has been shown that if TNAP enzyme is added to osteogenic media containing βGP, ectopic hydroxyapatite mineral deposition still occurs even in the absence of cells ^30^. To examine the effects of various mineralization substrates and agonists on metatarsal mineralization capability, we cultured E15 metatarsals in culture media containing a range of supplements, and images and length measurements were recorded daily. Results indicated that E15 metatarsal bones still grow and mineralize their ECM in the absence of βGP. E15 metatarsal bones cultured in ascorbic acid only media showed comparable increases in length and mineralization as metatarsals cultured in the presence of βGP (**Figure 3**).

We have also shown that lentivirus may be used to transfect exogenous DNA into cultured metatarsals. Here we successfully transfected metatarsal bones on day 0 of culture (*i.e.* day of dissection) with 2 x 10^6^ green fluorescent protein (GFP) virus particles per metatarsal. To enable virus transduction, polybrene was simultaneously added to cultures at 1:500. Cultures were left for 7 days, with no media changes as required for E15 metatarsal mineralization to occur, before being incubated with 4',6-diamidino-2-phenylindole (DAPI) stain for 5 min and then directly visualized using a confocal microscope (**Figure 4**). Our proof of concept experiments clearly show effective transduction. However it would be prudent to examine sections throughout the entirety of the metatarsal bone to assess their transfection and effective gene manipulation.


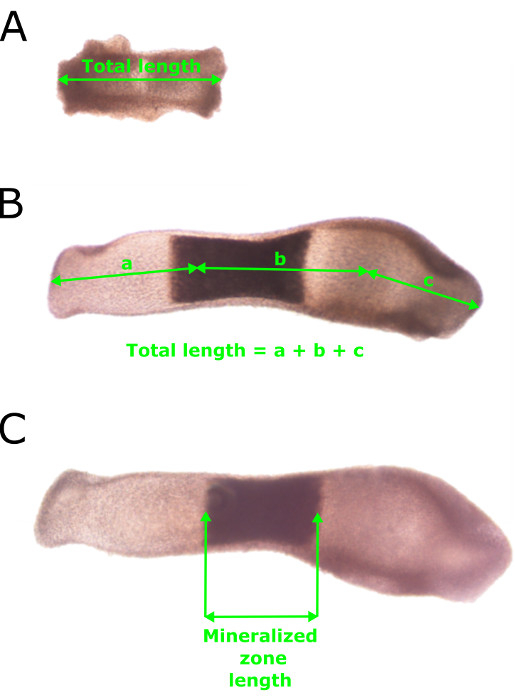
**Figure 1:Standard Methodology used to Measure the Length and Mineralization Zone of Embryonic Metatarsals.** (**A**) Total length measurements of E15 metatarsals on day 0 of culture (day of dissection) taken through the center of the metatarsal. (**B**) Measurements of total longitudinal length of a metatarsal after seven days in culture. Note the slight curvature in the metatarsal. (**C**) Measurement of the mineralization zone length after seven days in culture. Please click here to view a larger version of this figure.


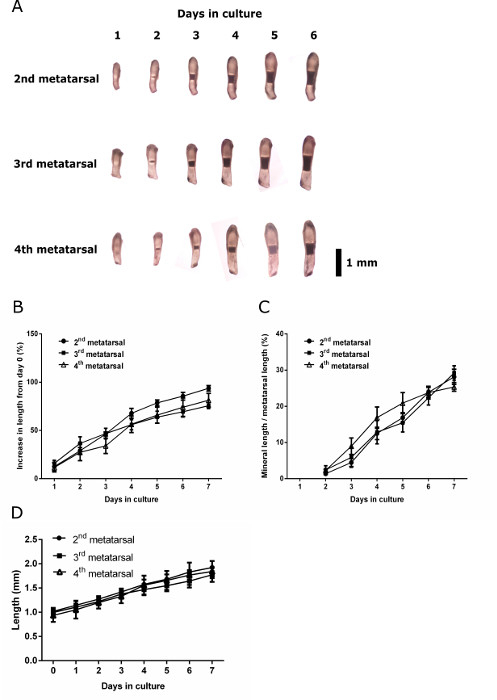
**Figure 2:Growth and Mineralization Capacity of Anatomically Distinct E15 Metatarsals.** Imaging and measurement of the 2nd, 3rd and 4th metatarsals from the hind limb of E15 mice was performed. (**A**) Serial images of the 2nd, 3rd and 4th metatarsals throughout the culture period. (**B**) Percentage increase in length from day 0 at each day of culture. (**C**) Mineral length of the 2nd, 3rd and 4th metatarsals over the culture period expressed as a percentage of the total metatarsal length. Data in are represented as mean ± standard error of the mean (S.E.M), (n = 6). (**D**) C57Bl/6J metatarsal lengths at each day of culture. Data in are represented as mean ± standard deviation (n = 6). Black bar = 1 mm. Please click here to view a larger version of this figure.


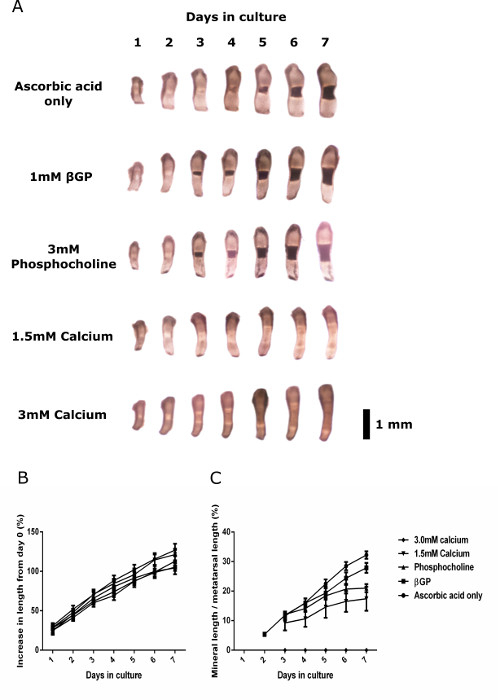
**Figure 3:Growth and Mineralization Capacity of Wild-type E15 Metatarsals Cultured in various Osteogenic Media.** (**A**) Serial images of metatarsals cultured in ascorbic acid only, 1 mM βGP, 3 mM phosphocholine, 1.5 mM calcium chloride or 3 mM calcium chloride containing media. (**B**) Percentage increase in length from day 0 of metatarsals cultured with the various supplements. (**C**) Mineral length of metatarsals cultured in various media expressed as a percentage of the total metatarsal length. Data are represented as mean ± standard error of the mean (S.E.M), (n = 6). Black bar = 1 mm. Please click here to view a larger version of this figure.


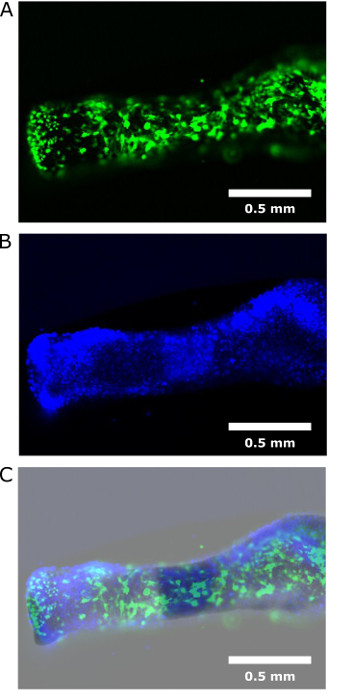
**Figure 4:Transfection of E15 Metatarsals with GFP Virus Particles.** (**A**) E15 metatarsal bones were transfected with GFP virus particles for proof of concept. (**B**) Bones were stained with DAPI for DNA visualization. (**C**) Dual images indicated successful transfection of GFP virus throughout the entirety of the metatarsal bones. White bars = 0.5 mm. Please click here to view a larger version of this figure.

## Discussion

The culture of murine embryonic metatarsals provides a highly physiological model of endochondral growth and mineralization. Early studies have validated that E15 mouse metatarsals undergo a normal pattern of skeletal growth and differentiation. The cartilage (chondrocytes) and bone (osteoblasts and osteoclasts) cells and their respective collagenous matrices are indistinguishable from those observed *in vivo*. However, there are some recognized limitations of this model. The speed of osteoclast differentiation is impaired as is bone growth (but not cartilage differentiation). Bone growth is approximately 50% slower than that observed *in vivo *and may be due to deficiencies in systemic factors which influence the production of local factors (*e.g.*, IGF-1, BMPs, *etc.*) ^31^. Also, it is well recognized that bone is sensitive to its mechanical environment and this is also the case for cultured metatarsals, which respond to changes in mechanical loading ^32,33^. Therefore, in unloaded situations, as described in this protocol, mineralization and mineral resorption may be compromised. Nevertheless, the metatarsal model offers the ability to directly measure linear bone growth which is not possible via 2D and 3D *in vitro *culture systems.

We have provided a comprehensive description of the protocol involved in the dissection and culture of E15 murine metatarsals and indeed, for the first time, confirm the validity of pooling the 2^nd^, 3^rd^ and 4^th^ metatarsals for experiments.

Metatarsals from E17/E18 are commonly used to investigate the mechanisms of bone growth owing to their extraordinary potential to grow *ex vivo* for long periods of time (*i.e. *beyond 14 days in culture). They are a well-established model to investigate the roles of growth factors on embryonic longitudinal bone growth. Additionally, the dissection and culture of postnatal metatarsals is commonly employed to delineate the mechanisms surrounding postnatal bone growth as it is understood that postnatal bone growth and fetal bone growth are regulated differently ^21^. The bone growth observed in cultured postnatal metatarsals is however significantly less than that observed in embryonic rudiments ^25,34^, limiting their potential in long term studies and highlighting the more systemic influence on bone growth at this postnatal stage. Indeed, 3 day old postnatal metatarsals from mice are typically the latest stage that can be used to obtain measurable growth ^34^.

Here we describe our protocol for the isolation of embryonic metatarsal bones at E15. The absence of hydroxyapaptite mineral in E15 metatarsal bones means that they provide an unrivaled model to investigate not only the mechanisms surrounding longitudinal bone growth, but also the initiation of skeletal mineralization. The mineralized matrix of the terminal hypertrophic chondrocyte zone provides a scaffold for invading osteoblasts to lay down a bone specific matrix (osteoid) which is subsequently mineralized, and as such this initial mineralization is vital for successful and functional bone formation ^35^. Indeed a number of recent reports utilizing E15 metatarsals have confirmed their unique ability for the investigation of the mineralization process ^28,36,37^. In addition, researchers have described the use of E15 metatarsals as a model of angiogenesis ^38^, highlighting the potential of embryonic metatarsals as model outside the bone growth/mineralization setting.

The protocol we describe requires only standard laboratory equipment including a laminar flow hood, a dissecting microscope for performing the dissection, and a CO_2_ incubator for the culture of excised rudiments. Furthermore, a very basic culture medium containing αMEM, BSA, antibiotics, antimycotics and L-ascorbic acid (a co-factor in the synthesis of hydroxyproline and hydroxylysine, two essential amino acids for the production of collagen ^39^) avoids the use of undefined additives, such as animal sera. Traditionally, investigators have added βGP to media used to culture embryonic metatarsals but as revealed in our results, the use of an additional phosphate source is not required for successful mineralization in this culture system. This is an important finding in our pursuit of understanding the mechanisms underpinning ECM mineralization.

Metatarsals have previously been infected with adenoviruses containing dominant-negative forms of Smad2 to explore the role of transforming growth factor β in the regulation of long bone development ^40^. Here we also reveal the successful transfection of wild-type E15 metatarsal bones with GFP virus particles, indicating that lentiviral techniques could easily be adopted to manipulate gene expression in metatarsal cultures. Similarly, the metatarsal organ culture system is an excellent model in which to compare the growth of bones from genetically altered mice to better understand the role of a particular gene in the bone growth process. Our previous studies have utilized the metatarsal organ culture system to determine the role of suppressor of cytokine signalling-2 (SOCS2) in endochondral bone growth. Using metatarsal bones from mice deficient in SOCS2, we have shown that growth hormone is able to simulate their longitudinal growth independent of insulin like growth factor (IGF-1), unlike wild-type metatarsal bones which do not respond to growth hormone treatment ^34,41^. This highlights the extent to which metatarsal cultures can be manipulated to examine the effects of different genes and/or exogenous factors on endochondral bone growth.

In summary, we have detailed a method for the successful extraction and culture of embryonic metatarsal bones which may be manipulated and examined using a variety of analyses. This *ex vivo* model maintains cell-cell and cell-matrix interactions, as well as contains chondrocytes in different phases of chondrogenesis, therefore providing a more physiological model than cells in monolayer or 3D culture. Metatarsal organ cultures are therefore a unique model for investigating the molecular mechanisms responsible for endochondral ossification, and are essential to further our understanding of both bone physiology and pathophysiology

## Disclosures

The authors have nothing to disclose.
